# Drivers for low-value imaging: a qualitative study of stakeholders’ perspectives in Norway

**DOI:** 10.1186/s12913-023-09328-4

**Published:** 2023-03-28

**Authors:** Ingrid Øfsti Brandsæter, Eivind Richter Andersen, Bjørn Morten Hofmann, Elin Kjelle

**Affiliations:** 1 Department of Health Sciences Gjøvik, Norwegian University of Science and Technology (NTNU), NTNU Gjøvik, PB 191, 2802 Gjøvik, Norway; 2 Centre for Medical Ethics, University of Oslo, Blindern, PB 1130, 0318 Oslo, Norway

**Keywords:** Low-value imaging, Diagnostic imaging, Health services misuse

## Abstract

**Background:**

One kind of overutilization of diagnostic imaging is low-value imaging, i.e., imaging that does not lead to altered clinical pathways or improved health outcomes. Despite having well-documented extension and consequences, low-value imaging is still widespread. The objective of this study was to identify the drivers for the use of low-value imaging in the Norwegian healthcare services.

**Methods:**

We conducted individual, semi-structured interviews among representatives from the health authorities, general practitioners, specialists working in hospitals, radiologists, radiographers, and managers of imaging departments. Data analysis was carried out in line with framework analysis consisting of five steps: Familiarization, indexing, charting, mapping, and interpretation.

**Results:**

The analysis included 27 participants and resulted in two themes. The stakeholders identified drivers in the healthcare system and in the interaction between radiologists, referrers, and patients. The identified drivers were categorized in sub-themes, such as organization, communication, competence, expectations, defensive medicine, roles and responsibilities, and referral quality and time constraints. The drivers interact with each other and may strengthen the effect of other drivers.

**Conclusions:**

Several drivers for low-value imaging in Norway were identified at all levels of the healthcare system. The drivers work simultaneously and synergistically. To free resources for high-value imaging, drivers should be targeted by appropriate measures at several levels to reduce low-value imaging.

**Supplementary Information:**

The online version contains supplementary material available at 10.1186/s12913-023-09328-4.

## Background

Imaging is an important part of modern medicine and is essential for effective and efficient patient management at all levels of healthcare [1]. However, the utilization of diagnostic imaging varies as seen in the USA [2–6], Europe [4, 7], and Norway specifically [8–11]. The variation indicates an uneven distribution of resources.

Overutilization of healthcare services may be caused by low-value care, defined as “an intervention where evidence suggests it confers no or very little benefit on patients, or risk of harm exceeds likely benefit, or, more broadly, the added costs of the intervention do not provide proportional added benefits” [12]. Low-value imaging is estimated to account for 20‒50% of all imaging internationally [1, 13]. Internationally, several low-value examinations have been identified, for example, imaging of lower back pain, brain imaging in minor head injury, and various types of routine imaging, as these examinations often do not improve patient outcomes, compared to if the examination were not performed [14–16].

Low-value imaging has several potential negative consequences. In addition to representing waste, medical expenditures, and alternative costs [17], imaging entails several risks for patients, such as radiation exposure [18], false (positive or negative) test results, incidental findings which may lead to further examinations and unnecessary treatment [19], and negative side effects, such as from the use of contrast agents [20]. Risks are related to all types of imaging modalities, such as conventional x-ray, computed tomography (CT), and magnetic resonance imaging (MRI).

Earlier research has identified several drivers for low-value care and low-value imaging, ranging from system-level drivers such as financial incentives and time constraints, to individual-level drivers such as patients’ expectations and defensive medicine [1, 21–25]. However, drivers are contextual, and to our knowledge, there are no previous studies on the drivers for low-value imaging in Norway. This study aims to add to existing knowledge by identifying the drivers for low-value imaging in Norway. The objective of our study was to identify stakeholders’ experience of drivers for use of low-value imaging in the Norwegian healthcare services and their perspectives on these.

## Methods

This qualitative, semi-structured interview-based study reports on participants’ perceptions of drivers for low-value imaging, using a large dataset collected in Norway. A study on the same participants’ perceptions of suitable measures for reducing low-value imaging has already been published [26], thus a supplementing description of the data collection method can be found there.

### Context

The healthcare system in Norway is divided into primary and specialist healthcare. Primary healthcare includes care services, rehabilitation and social services, and is organised by the municipalities. Specialist healthcare consists of hospital trusts managed by regional health authorities. The healthcare services are mainly public, and funded by general taxation, while patients only pay a small user fee per service. The financing of the healthcare system, including radiological services, is partly covered by reimbursements from the government. The reimbursement is given as a fee per service reimbursed to the organization and not the individual health care provider [27].

In addition, there are private imaging centres in urban areas. Some of these centres are partly commissioned by the health authorities to cover specific tasks. Nevertheless, it is possible for patients to gain quicker access to imaging with out-of-pocket payment or through personal health insurance policies. Whether the patient is going to attend a public or private imaging centre, a referral is required from a doctor, manual therapist, or chiropractor [27]. The Norwegian radiation protection regulations state that all referrals should contain sufficient information to assess the justification of the examination for the specific patient [28]. The receivers of referrals are the imaging departments in hospitals or private imaging centres. The appropriateness of referrals is confirmed by a radiologist or, in some private imaging centres, by a radiographer.

### The research team

This study was conducted by a multi-professional team of researchers: IØB and ERA – radiographers and PhD-candidates, and EK (radiographer/postdoc) and BMH (natural scientist/philosopher/professor) both experienced researchers, in addition to AMK (medical doctor) – see acknowledgements.

### Recruitment

Recruitment letters were sent to hospitals and municipalities, seeking permission to conduct the study, and identifying a contact person to help with recruitment. Potential informants were identified in collaboration with the contact person and invited to participate by email. This included an attached information letter and consent form. One participant was directly contacted due to expertise in the area. Nested strategy led to the inclusion of two more participants.

The plan was to conduct 30 interviews (5 radiologists, 5 radiographers, 6 managers, 5 hospital clinicians, 5 general practitioners (GPs), and 4 health authorities’ representatives). When an adequate number of interviews had been conducted, the recruitment process was halted.

### Data collection

A semi-structured interview guide was developed, pilot tested, and applied (Additional file 1), see Andersen et al. [26] for more details. Data collection was performed by ERA and AMK.

The interviews started with a formal introduction providing the same information to every participant. To obtain a rich material, the interview guide was not followed strictly. However, it was used to ensure that all topics and questions were covered throughout the session.

The interviews were conducted by video call (Zoom Video Communications, Inc., San Jose, USA) due to the COVID-19 pandemic, between February and June 2021.

### Data management and analysis

The interviews were transcribed verbatim by ERA, AMK, EK and a transcriber. Data analysis was performed in line with framework analysis as described by Ritchie and Spencer [29, 30], consisting of five steps: familiarization, indexing, charting, mapping, and interpretation. A description of the steps and the action taken by the research team is presented in Table 1. An example of the analysis, from summary to categorization, can be seen in Table 2.

**Table 1:** Steps and contribution in the framework analysis

Step	Description of step	Action
Familiarization	• Reading the transcripts • Creating a preliminary framework based on some transcripts • Testing and adjusting the framework against new transcripts	All authors read the transcripts. In addition, IØB and BMH listened to the recording of several interviews ERA, AMK and EK subsequently discussed the content of the transcriptions and created a preliminary framework The framework was further adjusted in light of further transcriptions by adding transcriptions by ERA, EK, AMK and BMH The framework consisted of 7 categories and 84 codes (Additional file 2) [26]
Indexing	• Indexing data relevant to the objective according to the framework	IØB and ERA indexed interviews 1‒6 separately according to the framework, and discussed the indexing practice until a consensus was reached. The team (IØB, EK, ERA and BMH) discussed the indexing on two occasions IØB indexed all transcripts using NVivo software (release 1.6.1, QSR international Pty Ltd., 2022)
Charting	• Making summaries of the indexes • Creating matrixes with summaries: participants in rows, and the indexes in columns • Refining the framework according to the matrixes	Summaries of the indexed data were placed in matrixes consisting of the participants in rows, and the indexing in columns. All authors discussed and rearranged the matrixes, resulting in a new, condensed framework The new framework consisted of 3 categories and 21 codes (Additional file 3)
Mapping	• Reorganising the summaries in line with the new framework • Identifying key elements based on summaries • Grouping key elements into key dimensions • Creating categories from key dimensions	All summaries were reread and reorganised by IØB Key elements were identified and sorted by IØB IØB and EK extracted key dimensions based on the key elements All authors discussed the content in all mapping steps, creating categories from the key dimensions
Interpretation	• Discussing the meaning of the content in the categories • Refining the categories • Looking for linkages between categories • Checking findings against the transcripts	All authors participated in the interpretation, discussed and refined the categories by reorganizing key dimensions and by looking for linkages between categories IØB verified the findings against the transcripts

**Table 2:** An example from the analysis

Summary	Key elements (elements detected in the data)	Key dimensions (broader, more refined categories)	Categorization
It is no longer sufficient for the patient to only show clinical improvement. It must be verified by diagnostic imaging as well. Referrers have expectations and need images	-Not enough with clinical improvement -Referrers expect and need images	-Clinical examination -Just to be safe	Defensive medicine

### Ethics

Participation was voluntary, and informed consent was obtained from all participants. The processing and storage of personal information was approved by the Norwegian Centre for Research Data, approval number 475812.

## Results

Twenty-seven participants from six different occupational groups (see Table 3) were included in this study. Several of the participants labelled as “managers” were radiologists working as managers in an imaging department. The participants worked in regional or local hospitals, medical offices, private imaging centres, and the public sector. Four participants were representatives from the Norwegian health and radiation protection authorities. Half of the participants worked in urban areas, while the other half worked in rural areas/local hospitals. The mean length of the interviews was 59 min (37‒181 min).

**Table 3:** Participants’ occupational group and number of participants in the groups

Occupational group	Details	N participants
Authorities	Health authorities	2
	Norwegian Radiation and Nuclear Safety Authority	2
General practitioner	Public general practitioner office	5
Specialist	Orthopedist	1
	Oncologist	1
	Neurologist	1
	Medicine	2
Radiologist	Hospital	3
Radiographer	Hospital	3
	Private imaging centre	1
Manager	Hospital	5
	Private imaging centre	1
Total		27

**Fig. 1:**
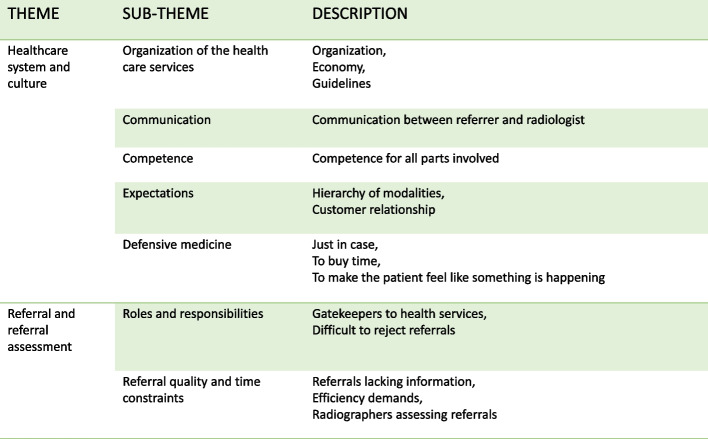
Overview of themes and subthemes

The analysis resulted in two themes; *Healthcare system and culture* and *Referral and referral assessment,* with several subthemes (see Fig. 1). The results are presented according to these themes. All participants identified several drivers of low-value imaging, on different levels of the healthcare system. One radiologist said:

“*I think that there are many drivers, and they exist on different levels. They are with the patient, with the families, with the referrers, and with the radiologists”*

### Healthcare system and culture

#### Organization of the healthcare service

Most participants expressed the opinion that the organization of the healthcare services is a driver for low-value imaging. Easy access to imaging, both the patient access (distance to a radiological department) and the number of service availability (modalities) were considered a driver as accessibility increases use. However, participants emphasised that there are great geographical differences in Norway – in the northern part of Norway and in other rural areas, the public hospitals are smaller, and there are fewer private imaging centres compared to the southern/eastern part of the country. One GP said:

*“The first years as a GP, I worked on an island, far off the beaten track (…). Back then, I preferred to see the patient the next day to see how the patient is coping, because it was a day’s journey to get an X-ray. Whereas where I work now, it’s only one hour from a place they can get an X-ray, so of course I use it much more now”*

Economic incentives were also mentioned as a driver by all occupational groups as there are financial incentives to perform examinations, but no incentives to refuse an examination. However, some referrers, managers and radiologists, stated that they do not think about economics in their everyday work. Also, private health services were described as a driver for low-value imaging. Several participants agreed that private imaging centres do more unjustified examinations than public hospitals. On the other hand, private imaging centres were positively described as highly efficient and able to do more examinations in one day compared to public hospitals. A participant from the health authorities claimed that there was the same amount of low-value imaging in private centres and public imaging departments. One manager/radiologist stated:

“There will always be people who make choices with medical consequences (…) where some of their motivation is something other than medical, for example financial incentives”

Participants described the national diagnostic pathways developed by the health authorities as a driver of low-value imaging. Also, national guidelines were described a driver for several reasons. First, radiologists stated that they are not always involved in making guidelines for various disciplines, hence the medical indication to carry out imaging is not always present in guidelines. Second, the interpretation of guidelines varied among the participants. Some considered guidelines as recommendations, with room for manoeuvre within a guideline, while others felt bound by them and believed there should be a good reason to deviate from them. Third, leaders and specialists also stated that guidelines could justify less warranted radiology. As long as guidelines are followed, no one can blame you for a mistake later. One specialist stated:

*“Guidelines can be a clear driver for radiological examinations”*

On the other hand, it was suggested that a lack of guidelines, especially on writing referrals, increased the use of low-value imaging.

Local protocols and routines in the different hospitals and private imaging centres can lead to low-value imaging. Since various imaging departments have specific protocols, patients often need a retake if the images are requested at another hospital. In addition, routines differ in hospital departments. Participants emphasised that the healthcare system are organized in ways making healthcare professionals act in a siloed manner. As such, health professionals’ concerns only about their own areas of expertise and their patients. Thus, providers from various hospital departments could make choices affecting other departments without acknowledging the consequences. One manager/radiologists gave this example:

*“Chest X-ray has always been misused, almost as an administrative tool. I used to work in a hospital where we were told by the specialty registrars in internal medicine that they had learned to order a chest X-ray to get a quick and effective overview of all new patients”*

### Communication

Communication challenges were considered a driver of low-value imaging, as radiologists and radiographers found it difficult and time-consuming to contact GPs and specialists in the hospital. Participants agreed that dialogue between referrers and the imaging department was important, and that lack of communication could be a driver for low-value imaging. The specialists (working in a hospital) stated it was easy to contact radiologists, while GPs experienced difficulties in reaching the radiologists and rarely received feedback on referrals. One radiologist said:

*“We do not have an effective communication channel with the referrers. That applies to both internal [in hospitals] and external referrers. That’s probably the most frequent reason why referrals don’t get rejected”*

Furthermore, several occupational groups experienced the electronic communication system as inadequate. Without a national shared image archive, the system lacks information about patients’ examinations elsewhere. Additionally, the method used to send images from one imaging department to another, especially between private imaging centres and public hospitals, was described as challenging. One manager/radiologist said:

*“We do not have a shared image archive (…). If we had access so we could see which [examinations] patients have done around the country when they are referred, we would have avoided a lot of unnecessary diagnostic imaging”*

### Competence

Participants from all occupational groups mentioned a lack of competence as a driver for low-value imaging. A multi-professional right to refer patients to imaging, applying also to chiropractors and manual therapists, was believed to lead to both over- and underutilization. Radiologists stated that the referrers do not possess enough knowledge about the different modalities and the consequences of imaging. However, it was thought that experienced referrers often provide referrals of better quality. Participants described a poor evidence base for the imaging of various patient groups as a reason for low-value imaging, leading to inappropriate diagnostic imaging and a higher frequency of follow-ups than necessary. Furthermore, the lack of knowledge was reported to lead to excessive confidence in the value of imaging and technology and described as a driver for low-value imaging, as images are used to visualize the problem, regardless of the patient’s issue. One specialist said:

*“I think that the referrer does not know that [MRI] is a poorer examination for the purpose [arthrosis], but thinks that the modality that gives the best overview of anatomy in general also gives the best overview of pathology”*

### Expectations

Most participants described expectations from patients and their families as a driver for low-value imaging. They expressed the view that patients and their families often believe that an image can solve their health problems, thus pressuring their GP to refer to MRI or CT. The participants believed that in addition to the referrers, the general population have an exaggerated belief in diagnostic imaging and that there is a hierarchy of modalities, with MRI on top.

“Expectations are a driver. In other words, the population, both referrers and patients expect aggressive examination and monitoring”

Managers and representatives from the health authorities said that GPs should be better at saying ‘no’ to patients who demand images. Also, GPs and specialists considered it essential to achieve a common understanding with the patients. Being honest with the patient about risks such as incidental findings or over-diagnosis, was considered important. Patients often accept the GP/specialist’ decision to postpone or reject imaging if they are provided with a good reason. However, such time-consuming efforts makes it difficult to say ‘no’ when patients ask for MRI or CT. On the contrary, a specialist believed referrers have other reasons for not rejecting the demand from the patients, and said:

“One reason [for the referrer] to refer to imaging (…) could be to buy time. The patient will experience that something is happening.

The relationship between patient and doctor was described as a customer relationship, where GPs are afraid of losing the patient to another GP who would refer the patient anyway. Furthermore, GPs described their fear of losing their good standing and spoiling the relationship with their patient if they do not meet patients’ requests. Therefore, imaging is sometimes used in negotiations with patients to build trust. A representative from the health authorities said:

“GPs are afraid of losing their customers. These are not just patients, they are also their customers”

### Defensive medicine

Participants identified elements of what has been called defensive medicine as a driver for low-value imaging. This includes doctors being afraid of making mistakes and ordering examinations ‘just to be on the safe side’ in order to buy time, or to make the patient feel like something is happening. This driver differs from the driver labelled as “expectations” as defensive medicine represent the heath professional’s inner uncertainty, while “expectations” is about external expectations. Signs of defensive medicine mainly originated from the (in)ability to handle uncertainties. Some participants stated that facing and handling uncertainties is a part of being a healthcare professional, while others said that there is little acceptance for making mistakes. All occupational groups said that fear of overlooking something serious made them use imaging ‘just in case’, even when they believed nothing was wrong. The fear of ending up on the front page of the newspapers was considered to be a driver. One GP said.

*“The 0-point something percent chance of overlooking a rare tumour trumps the probability of a false positive finding, or low incidence, and the fear of overtreatment it leads to. Nevertheless, it appears more important to rule this out. And, if you get a false positive, you still get a pat on the back, you are the hero after all. There is a terrible asymmetry there”*

Several participants pointed to reduced trust and less use of clinical examinations as drivers for low-value imaging, and asserted that clinical assessment has a low standing. Low-value imaging could have been avoided if the clinical examination was better or if the GP had trusted their clinical judgment. When asked why low-value examinations are referred, a specialist answered:

“One has an inherent tendency to trust an image more than the clinical assessment and one’s own knowledge”

### Referrals and referral assessments

#### Roles and responsibilities

The participants acknowledged that health professionals have different roles and responsibilities in healthcare services, but they disagreed on the content of these roles and responsibilities. Both referrers and radiologists described themselves as a gatekeeper to health services. Some GPs considered themselves responsible for choosing the appropriate examination as they know the patient history, while radiologists felt responsible for assessing justification of the examination and selecting the suitable imaging modality. However, radiologists considered this assessment difficult as they do not know the patient as well as the referrer. Radiologists experienced difficulties in rejecting referrals to examinations deemed as low-value. One radiologist said:

*“The referrer is very important for us radiologists because we have an obligation to assess all referrals we receive. It is clear that we are allowed to reject them or change the requested modality, but the threshold for rejecting is relatively high as we respect that the referrer knows the patient and has the total picture”*

#### Referral quality and time constraints

The radiologists and radiographers described referrals lacking relevant information or uncertain indication as challenging to evaluate, and thus driving low-value imaging. They stated that more information in the referrals is necessary to make the best choice of imaging modality and procedure. Furthermore, radiologists described referrers as leaving out essential information or adding false tentative diagnoses to bring the examinations forward. Nevertheless, such referrals were often not rejected due to the risk of being seen as difficult. One radiographer said:

*“In a sense, a ‘no’ has only negative consequences. You get an irritated referrer, an irritated patient, lost income, and you may miss [diagnosing] a disease. There is almost nothing positive about saying no”*

However, managers and a GP stated that rejections have a learning effect.

Moreover, the rejection of referrals was perceived as difficult due to efficiency demands. It felt easier and more time-efficient to accept referrals whether the examination was indicated or not. For example, the same referral was often re-referred, either as is or with additional information, and would then be accepted. Hence, the initial rejection only resulted in additional work for both the radiologist/radiographer and the referrer. One radiographer stated:

*“Sometimes it can be easier to image than to spend a lot of time getting hold of additional information. On occasion we send referrals back, and then they are returned with a bit more information, and then it [the examination] will be taken anyway”*

In some imaging departments, radiographers assess referrals based on efficiency demands and time constraints. A radiographer acknowledged that referral assessment is a subjective evaluation, which is always difficult. Additionally, a manager said that radiographers had a higher threshold for rejecting referrals and this is a driver of low-value imaging. One manager/ radiologist said:

*“Ideally, there should be enough capacity and resources for us [radiologists] to assess absolutely all referrals. However, it costs a lot of money. And when there are too few radiologists and a long report time, it becomes a trade-off.”*

## Discussion

**Fig. 2:**
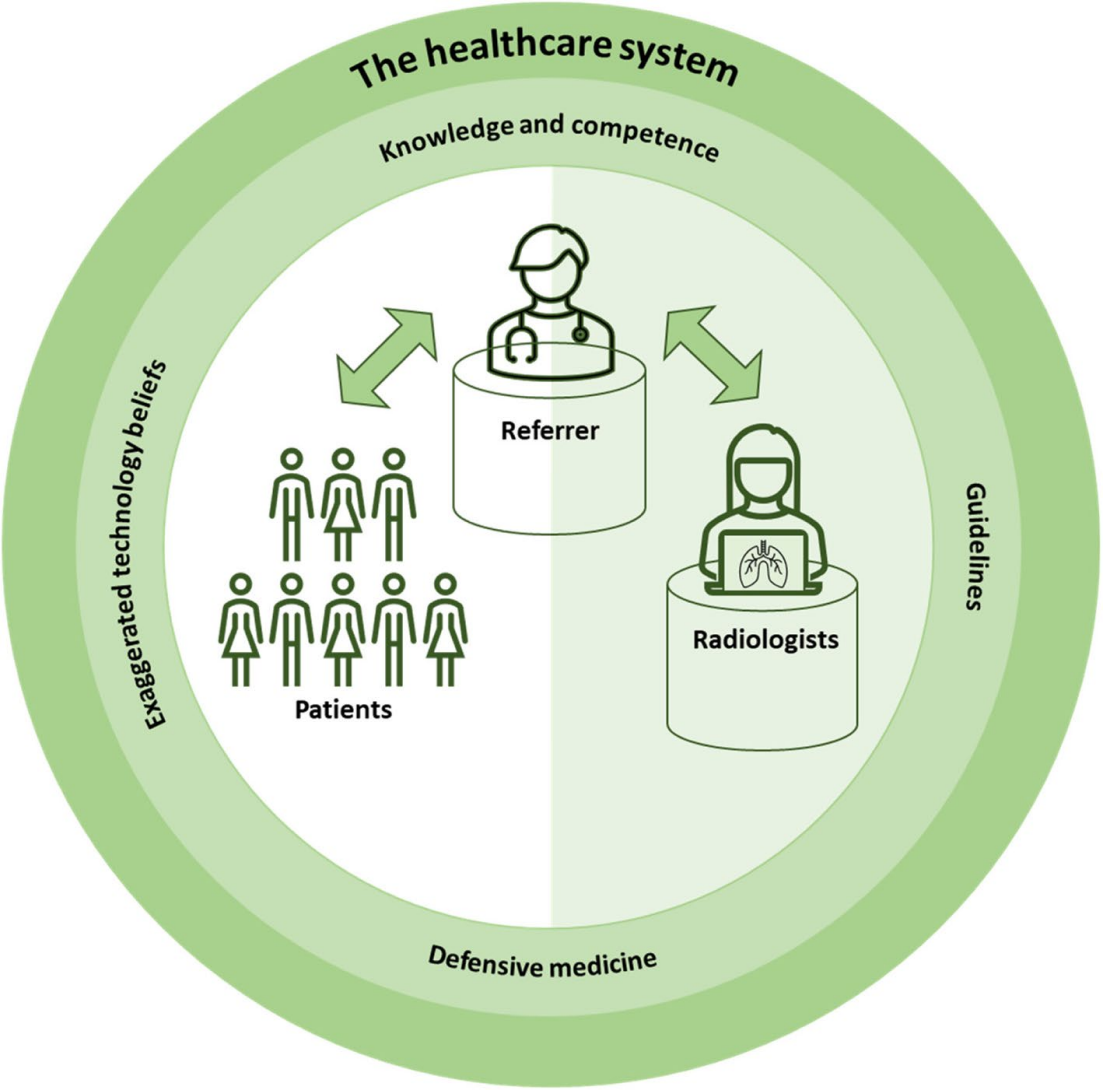
Drivers for low-value imaging in the Norwegian healthcare system. The outer circle in the figure illustrates the drivers in the organization of the healthcare system as economy, and good access to imaging. The middle circle illustrates the procedural and cultural drivers. The inner circle represents the process of referral and referral assessment. The referrer and the radiologist are placed in separate silos, and the split demonstrates that the referrer is the only connection between the patients and the radiologists

In this study we aimed to identify drivers for low-value imaging in the Norwegian healthcare system. The stakeholders identified drivers both in the healthcare system and in the interaction between radiologists, referrers, and patients. Figure 2 visualizes the drivers identified in this study.

### The healthcare system

An important finding of this study was how the organization of the healthcare system led to drivers for low-value imaging such as access to imaging services, the understanding of different roles and their responsibilities, silo mentality, and economic incentives. The healthcare services' overarching structure (as illustrated in the outer circle in Fig. 2) was an important premise for individual drivers of low-value imaging. It leads to a silo mentality where clinicians working in one department in a hospital think only about their own patients and resources, not the consequences their actions might have on other departments. For example, sending an inappropriate referral was perceived to be easier than spending time with the patient and discussing other alternatives.

As the healthcare services are designed for quick and effective work, participants reported that time constraints are an important driver for low-value imaging, a finding which is supported by other healthcare studies [25, 31, 32]. In addition, the financial system drives efficiency as GPs or imaging centres/departments are reimbursed per patient or per examination. However, participants highlighted that there are no economic incentives to avoid examinations and no economic benefits from spending extra time with the patient to explain why imaging may be unnecessary. Nevertheless, while some participants considered economic incentives as a driver for low-value imaging, others stated that the reimbursement from the governments barely covers the expenses of a referral, thus should not be regarded as a driver. In the literature, economic incentives are considered a driver for overutilization, as the structure promotes volume over value [33, 34]. Participants, especially those who worked in public hospitals, considered the private imaging centres to be most affected by economic incentives.

In addition, private imaging centers were considered to contribute to better access to imaging. However, good access, in terms of short travel distances and high equipment availability are known drivers for the use of low-value imaging [35]. Some of the participants expressed a difference between the drivers for private and public providers, in terms of economic incentives and access. However, this was not very outspoken. One reason for this may be that the private providers are commissioned by the public health care system.

Access and use of services vary within the country [10, 11, 36], and the participants emphasized this geographical variation. Good access to imaging makes it challenging for the referrer to decline a patient’s request and for the radiologist to decline a referrer’s request. Interestingly, both GPs and radiologists in the present study consider themselves as gatekeepers for imaging services although radiologists do not have the time or enough information to act as gatekeepers [1].

### Culture and procedures

The middle circle of Fig. 2 illustrates procedural and cultural drivers. “Knowledge and competence” is placed at the top as this driver affects patients, referrers, and receivers. According to the participants, lack of knowledge is an important reason why patients requested examinations. In addition, patients and referrers may have an exaggerated belief in technology, and this is why the most advanced technology is often preferred, a finding which is supported by other studies [37–39]. Receivers also find it hard to reject examinations because they have little knowledge about the patient and the referrers' assessment.

GPs and specialists in this study stated that they often refer to imaging instead of trusting their own clinical judgment due to fear of the consequences of missing a serious condition and difficulties in handling uncertainty. Defensive medicine has been described as an important driver for overutilization for imaging, and also applies to other parts of the healthcare system [1, 31, 33, 34, 40].

Furthermore, some participants in the present study find it difficult to deviate from guidelines, stating that guidelines are drivers for low-value imaging. Interestingly, radiologists said that they are usually not involved in guideline development, resulting in what they feel are inadequate recommendations for imaging. In addition, the guidelines may be interpreted differently and result in referrals to low-value examinations, as supported by earlier research [32, 33]. On the other hand, guidelines are suggested as a measure for reducing low-value imaging as they can be used in the communication with patients. Hence, guidelines are a double-edged sword, and must be flexible and updated [26].

### The referral process and referral assessment

The inner circle of Fig. 2 illustrates the process of referral and referral assessment. Increased demands from the public and fear of losing patients to other health professionals resulted in GPs finding it hard not to meet the patient’s expectations, thus referring them to low-value examinations. Patients’ and their families’ requests and expectations for services have been identified as a driver for low-value care in several parts of the healthcare system [23, 24, 40, 41]. In addition, professionals have a desire to meet patients’ requests [31], but may misinterpret the patients’ expectations, assuming that the patient wants care without asking for it [34]. In the present study, the GPs found it helpful to talk with the patients about the risks and benefits of imaging, and to reach a common understanding of the situation. However, a lack of time for common decision making was described as a reason for not rejecting the request, in line with earlier research [24].

The referrer and the radiologist are placed in separate silos (Fig. 2), representing the previously mentioned silo mentality. Such a silo mentality without cooperation and effective information exchange seems to be a driver for low-value imaging. This finding corresponds with findings from other parts of the healthcare system, describing lack of communication as an important driver for low-value care [32, 42].

Surprisingly, some referrers expressed an expectation that radiologists should accept every referral unconditionally. However, according to the Norwegian radiation protection regulations, the radiology department is required to assess the justification of the referral [28]. Poor referral quality has been investigated in earlier studies, leading to difficulties in justifying the examination [43, 44]. Interestingly, there seem to be poor compliance with the statutory provision as the radiologists found it hard to reject referrals on account of poor referral quality, lack of knowledge of the patient, fear of stirring up the relationship with the referrer and fear of failing to identify severe illness.

### Implications for practice

All drivers identified in the present study work simultaneously and affect the use of low-value imaging. Verkerk et al. [34] found that the synergistic relationship between different factors strengthens the effect of other factors. Figure 2 illustrates how the different drivers work together, for instance, how the organization and financial system cause siloed manners by healthcare professionals, and poor communication between referrers and radiologists. Further research should include patients and other important stakeholders, and the relationship between different drivers needs to be explored. Measures for reducing low-value imaging must target several parts of the healthcare service, both at the organizational and the individual level at the same time. Measures should not focus on one driver only. To reduce low-value imaging, it is vital to target drivers by putting appropriate measures in place, and hence free up resources for high-value imaging.

### Strengths and limitations

This study demonstrates a number of strengths. First, we interviewed a broad range of stakeholders, including senders and receivers of referrals, managers, and representatives from the health authorities, thus collecting data from various levels in the healthcare system. Second, this is the first study conducted in a Norwegian context about drivers for low-value imaging. Third, all authors were involved in the whole analysis process, which is thoroughly described. In addition, the study is in line with the checklist of Tong, Sainsbury & Craig [45] for interviews and focus groups, thereby achieving trustworthiness and transparent reporting. This study was conducted in Norway, however, similar challenges may be seen in other European countries.

However, this study also has limitations. Not all relevant stakeholder groups were included, such as patients, chiropractors, and manual therapists. Thus, the study lacks some important perspectives on drivers of low-value imaging. Patients are very heterogeneous, and the focus of the study has been drivers. Moreover, chiropractors and manual therapists represent a small group of referrers. The interviews were conducted digitally and were therefore deprived of the positive aspects of a face-to-face interview. In addition, digital interviews resulted in some technical difficulties in respect of poor sound quality and internet access. However, the digital solution was considered efficient as participants got the opportunity to participate from their workspace. We planned to interview 30 participants, but the data collection ended with 27 interviewees. The research team concluded that a sufficient sample size was reached regarding the objective during the familiarization process, and inclusion was stopped at 27 due to a limited time frame.

## Conclusions

This study has identified several drivers for low-value imaging in Norway. Twenty-seven stakeholders in the Norwegian healthcare system were interviewed, and the analysis revealed drivers related to the healthcare system and culture as well as to referral and referral assessment. These drivers work simultaneously and synergistically on both an organizational and individual level. Accordingly, to free resources for high-value imaging, measures should be aimed at the health care system, work-related culture, and individual practices around referral and referral assessment simultaneously.

## Supplementary Information

**Additional file 1.**

**Additional file 2.**

**Additional file 3.**

## Data Availability

The datasets generated and analysed during the current study are not publicly available due to individual privacy conditions but are available from the corresponding author on reasonable request.

## Abbreviations

CT: Computed tomography
GP: General Practitioner
MRI: Magnetic resonance imaging
