# Supplementary material for: Drivers for low-value imaging: a qualitative study of stakeholders’ perspectives in Norway
Source: BMC Health Serv Res. 2023 Mar 28;23:295. doi: 10.1186/s12913-023-09328-4 (PMC10044073; doi:10.1186/s12913-023-09328-4)
Supplement: Supplementary file 1 — Additional file 1. [file 12913_2023_9328_MOESM1_ESM.pdf]

Additional file 1. Interview guide [1]

1. **Experiences**
   What are your reflections on the definition of low-value care? Can you provide some examples of examinations in your practice that will fall under this definition?

   Can you tell me about your experience with low-value radiology?

   What do you think are the most important reasons for using low-value examinations?

   How do you think the following impact on the use of low-value services?
   - Economics
   - Organisational structures
     - Systems
     - Environmental mechanisms (access to services, demands, expectations)
     - Imaging 'just to be sure'

   Have you tried to reduce the use of low-value radiology? If so, what did you do, and how did the measure work?
   - How was the measure received?
   - Why do you think it worked/ did not work?

2. **Possible future measures**
   In your opinion, what does it take to reduce the use of low-value radiology?

   What does it take for the measure to work?

   What can make measures unsuccessful?

   How do you think the measures will be received, for example by:
   a. Colleagues
   b. Patients/next of kin
   c. Others

3. **Elaboration**
   Is there anything else you think it is important for us to know or that you wish to elaborate?

1. Andersen ER, Hofmann BM, Kjelle E: **Reducing low-value radiological services in Norway –a qualitative multi-professional study on measures and facilitators for change**. *BMC Health Services Research* 2022, **22**(1):678.
