# Supplementary material for: Drivers for low-value imaging: a qualitative study of stakeholders’ perspectives in Norway
Source: BMC Health Serv Res. 2023 Mar 28;23:295. doi: 10.1186/s12913-023-09328-4 (PMC10044073; doi:10.1186/s12913-023-09328-4)
Supplement: Supplementary file 2 — Additional file 2. [file 12913_2023_9328_MOESM2_ESM.pdf]

Additional file 2: Original framework [1]

**1.0**	**Health policy and organization**
1.1	Politics
1.2	Laws and regulations
1.3	Government
1.4	Social economics
1.5	Organisation of healthcare services
1.6	Access to services
1.7	Capacity and prioritising
1.8	Resources
1.9	Logistics
1.10	Financial incentives
1.11	Guidelines and patient pathways
1.12	Routines
1.13	Roles and tasks
1.14	Time constrains
1.15	Patient economics
1.16	Justice
1.17	Dignity
1.18	Management
**2.0**	**Quality in health care services**
2.1	Value for patients and next of kin
2.2	Value for referrers
2.3	Value for society
2.4	Estimation of benefits vs. costs
2.5	Referral quality
2.6	Efficiency vs. quality
2.7	Risks
2.8	Quality indicators

**3.0**	**Knowledge and competence**
3.1	Knowledge
3.2	Competence
3.3	Lack of competence
3.4	Research
3.5	Evidence
3.6	Opportunity to stay up to date
3.7	Access to information
**4.0**	**Interaction and communication**
4.1	Dialogue between patient and referrer
4.2	Dialogue between hospital referrers and radiologists
4.3	Dialogue between GPs and radiologists
4.4	Dialogue between colleagues in the same discipline
4.5	Trust
4.6	Co-selection
4.7	Interaction
4.8	IT-systems
**5.0**	**Attitude and culture**
5.1	Prioritisation challenges
5.2	Expectations to the radiological department
5.3	Defensive medicine
5.4	Management of uncertainties
5.5	Examination hierarchy
5.6	Professional ambition
5.7	Market-driven thinking
5.8	Expectations and desires
5.9	Resignation
5.10	Awareness
5.11	Willingness to change
5.12	Discretionarily assessments

5.13	Healthcare professionals’ authority
**6.0**	**Measures for reducing low-value imaging**
6.1	Consciousness-raising
6.2	Better interaction
6.3	Better communication
6.4	Guidelines and patient pathways
6.5	Decision support
6.6	Internal invoicing
6.7	Dedicated imaging slots
6.8	Revisions
6.9	Common IT-systems
6.10	Referral assessment
6.11	Feedback/guidance
6.12	Rejections
6.13	Education and courses
6.14	Discussion groups
6.15	Await/make plans
6.16	Change the financial system
6.17	Change in management
6.18	Right to order imaging
6.19	Multimodal measures
**7.0**	**Measure characteristics /effects**
7.1	Process
7.2	Complexity
7.3	Respect/ownership
7.4	Targeted
7.5	Include relevant parties
7.6	Duration/effect
7.7	Simple and low resource-intensive
7.8	Awareness

7.9	Whom the measure should be directed to
7.10	How the measure is received/experienced
7.11	Enforceability

Reference

1. Andersen ER, Hofmann BM, Kjelle E: **Reducing low-value radiological services in Norway –a qualitative multi-professional study on measures and facilitators for change**. *BMC Health Services Research* 2022, **22**(1):678.
