# Supplementary material for: Drivers for low-value imaging: a qualitative study of stakeholders’ perspectives in Norway
Source: BMC Health Serv Res. 2023 Mar 28;23:295. doi: 10.1186/s12913-023-09328-4 (PMC10044073; doi:10.1186/s12913-023-09328-4)
Supplement: Supplementary file 3 — Additional file 3. [file 12913_2023_9328_MOESM3_ESM.pdf]

Additional file 3: New framework

**1.0**	**Health policy and organization**
1.1	Organisation of healthcare services
1.2	Financial incentives
1.3	Guidelines and patient pathways
1.4	Time constrains
1.5	Dignity
1.6	Roles and tasks
1.7	IT-systems
**2.0**	**Quality in the health care system**
2.1	Value for patients and next of kin/referrers
2.2	Estimation of benefits vs. costs
2.3	Referral quality
2.4	Competence
2.5	Dialogue between patient and referrer
2.6	Dialogue between referrer and radiologists
2.7	Interaction
2.8	Referral assessment
**3.0**	**Attitude and culture**
3.1	Prioritisation challenges
3.2	Defensive medicine
3.3	Examination hierarchy
3.4	Market-driven thinking
3.5	Expectations and desires (external)
3.6	Resignation (internal motivation/ attitude)
